# The *Tetragnatha kauaiensis* Genome Sheds Light on the Origins of Genomic Novelty in Spiders

**DOI:** 10.1093/gbe/evab262

**Published:** 2021-11-26

**Authors:** José Cerca, Ellie E Armstrong, Joel Vizueta, Rosa Fernández, Dimitar Dimitrov, Bent Petersen, Stefan Prost, Julio Rozas, Dmitri Petrov, Rosemary G Gillespie

**Affiliations:** 1 Berkeley Evolab, Department of Environmental Science, Policy, and Management, UC Berkeley, California, USA; 2 Frontiers in Evolutionary Zoology, Natural History Museum, University of Oslo, Norway; 3 Department of Natural History, NTNU University Museum, Norwegian University of Science and Technology, Trondheim, Norway; 4 Department of Biology, Stanford University, California, USA; 5 Departament de Genètica, Microbiologia i Estadística & Institut de Recerca de la Biodiversitat (IRBio), Universitat de Barcelona, Spain; 6 Villum Centre for Biodiversity Genomics, Section for Ecology and Evolution, Department of Biology, University of Copenhagen, Denmark; 7 Institute of Evolutionary Biology (CSIC—Universitat Pompeu Fabra), Barcelona, Spain; 8 Department of Natural History, University Museum of Bergen, University of Bergen, Norway; 9 Section for Evolutionary Genomics, The GLOBE Institute, Faculty of Health and Medical Sciences, University of Copenhagen, Denmark; 10 Centre of Excellence for Omics-Driven Computational Biodiscovery, Faculty of Applied Sciences, AIMST University, Kedah, Malaysia; 11 Central Research Laboratories, Natural History Museum Vienna, Vienna, Austria; 12 University of Veterinary Medicine, Konrad Lorenz Institute of Ethology, Vienna, Austria; 13 South African National Biodiversity Institute, National Zoological Garden, Pretoria, South Africa

**Keywords:** gene family, Araneae, arthropod, repeatome, hawai'i, transposable element

## Abstract

Spiders (Araneae) have a diverse spectrum of morphologies, behaviors, and physiologies. Attempts to understand the genomic-basis of this diversity are often hindered by their large, heterozygous, and AT-rich genomes with high repeat content resulting in highly fragmented, poor-quality assemblies. As a result, the key attributes of spider genomes, including gene family evolution, repeat content, and gene function, remain poorly understood. Here, we used Illumina and Dovetail Chicago technologies to sequence the genome of the long-jawed spider *Tetragnatha kauaiensis*, producing an assembly distributed along 3,925 scaffolds with an N50 of ∼2 Mb. Using comparative genomics tools, we explore genome evolution across available spider assemblies. Our findings suggest that the previously reported and vast genome size variation in spiders is linked to the different representation and number of transposable elements. Using statistical tools to uncover gene-family level evolution, we find expansions associated with the sensory perception of taste, immunity, and metabolism. In addition, we report strikingly different histories of chemosensory, venom, and silk gene families, with the first two evolving much earlier, affected by the ancestral whole genome duplication in Arachnopulmonata (∼450 Ma) and exhibiting higher numbers. Together, our findings reveal that spider genomes are highly variable and that genomic novelty may have been driven by the burst of an ancient whole genome duplication, followed by gene family and transposable element expansion.

Significance

Despite being one of the most charismatic animal lineages, progress on spider genome evolution lags due to the challenges in sequencing and assembling their genomes, which involve genome size and repeat content. Here, we sequence the genome of *Tetragnatha kauaiensis*, a spider endemic to Hawai’i, and compare it with other available spider genomes. We find variation in terms of repeats and transposable elements; expansions in gene-content associated with metabolism, sensory perception, and immunity; and wide variation of chemosensory genes and venom genes.

## Introduction

With nearly 50,000 described species (World Spider Catalog 2021), and dating back ∼350 Myr (Fernández et al. 2018), spiders (Chelicerata, Araneae) have conquered most terrestrial ecosystems, from the cold Arctic to arid deserts (Jackson and Cross 2011; Dimitrov et al. 2012; Garrison et al. 2016; Fernández et al. 2018). Spiders play a key role in terrestrial ecosystems regulating community dynamics as major arthropod predators (Herberstein and Wignall 2011; Wilder 2011), having evolved a diverse array of adaptive solutions, which include, a rich cocktail of venoms to neutralize prey (Binford 2001; King and Hardy 2013), a color palette essential for camouflaging, mimicking, and signaling (Oxford and Gillespie 1998; Croucher et al. 2013; Cotoras et al. 2016), and the ability to produce silk for spinning webs and subduing prey (Vollrath 1999; Garb et al. 2010; Sanggaard et al. 2014).

Despite the advances in spider ecology, evolution, and systematics, knowledge of spider genomes still lags relative to other taxa. Most of the available spider genomes are of poor quality, being highly fragmented (Garb et al. 2018) and lack a substantial part of the genome, with only three recent exceptions involving chromosome-resolved genomes (Escuer et al. 2021; Fan et al. 2021; Sheffer et al. 2021). Several factors contribute to the sparse availability of high-quality spider genome assemblies, including the lack of a model organism among spiders (sensu *Drosophila melanogaster* in flies and *Tribolium castaneum* in beetles) (Brewer et al. 2014), and the challenges associated with sequencing spider genomes, which are characterized by high AT-content, repeats, heterozygosity, and often large genome sizes (Garb et al. 2018). Focus on non-model organism genomes shows that increased taxon-sampling leads to an improved understanding of the diversity and function of molecular mechanisms across the tree of life (McGregor et al. 2008), as it overcomes the biases from the limited number of model taxa, and highlights the idiosyncrasies throughout the tree of life. Consequently, a better representation of spider genomes will certainly help understanding spider diversity and evolution (McGregor et al. 2008).

A systematic analysis of spider genomes has the potential to unveil the genomic foundation of spider evolution. For example, the detection of duplicate *Hox* clusters suggested an ancestral whole genome duplication in the common ancestor of modern spiders and scorpions (Arachnopulmonata; Schwager et al. 2007), and this evidence was later on confirmed by the first spider genomes (Clarke et al. 2015; Schwager et al. 2017; Leite et al. 2018). The implications of whole genome duplications may, however, be multifarious and complex (Ohno 1970). On one hand, genome duplication may act as a catalyst for molecular novelty. Under this framework, the retention of duplicated genes and other genetic components may act as “reservoirs of genetic variation,” through processes of gene neo- and sub-functionalization (Lynch and Force 2000), and be of use when organisms encounter novel selective pressures (Li et al. 2018; Nieto Feliner et al. 2020; Schmickl and Yant 2021). Considering the evidence for gene duplicates in spider genomes, including spidroins (silk genes) (Sanggaard et al. 2014; Clarke et al. 2015; Babb et al. 2017; Garb et al. 2018; Sheffer et al. 2021), venoms (Sanggaard et al. 2014; Gendreau et al. 2017; Haney et al. 2019), chemosensory (Vizueta et al. 2018, 2019; Vizueta, Escuer, et al. 2020) gene families may yield insights on phenotypic innovation and the adaptation to novel environments. On the other hand, because genome duplication leads to a significant re-organization of the genome, it may cause deregulation of gene-expression networks or unlock the epigenetic suppression of transposable elements, which may proliferate across the genome and result in decreased fitness for the organism—“the genomic shock hypothesis” (McClintock 1984; Choi et al. 2020). In such a scenario, one expects to find variation in transposable element proliferation across genomes, and ultimately a substantial variation of genome size. The proliferation of transposable elements may thereby underlie genome size variation in spiders, which ranges between 0.74 and 5.73 C values (0.7–5.6 Gb) (Gregory and Shorthouse 2003) (http://www.genomesize.com/ checked in April 15, 2021; values for: *Habronattus borealis*, *Tetragnatha elongata*, respectively). Comparisons between different genome assemblies may yield important insights on the prevalence of gene duplications, neofunctionalization, and transposable element dynamics across different lineages.

Here, we report a genome assembly of the Hawaiian spider *Tetragnatha kauaiensis* and place it in the context of currently available spider genomes to assess signatures of genome evolution across spider lineages (supplementary table 1, Supplementary Material online). To do so, we first explore the completeness and duplication rates across the spider assemblies. Considering the role of transposable elements in driving genome size variation, we also assess transposable element load in each genome. Third, we quantify the expansion and contraction of gene families (based on gene similarity metrics), and classify the function of these families using Gene Ontology (GO). Finally, we delve deeper into the identification and comparison of chemosensory, venom, and spidroin (silk) genes, studying duplicates in a phylogenetic context. Focus on these three categories is grounded on their central role to the survival and fitness of spiders, and benefits from extensive research, including hand curated genes and databases.

## Results

### The *Tetragnatha kauaiensis* Genome

The *T. kauaiensis* genome assembly has a size of ∼1.08 Gb, distributed along a total of 132,391 contigs, comprising 3,925 scaffolds. The largest scaffold is ca. 10.5 megabases (Mb), whereas the estimated scaffold-N50 for the assembly is ∼2 Mb (supplementary table 2, Supplementary Material online). The assembly has a GC content of ∼33.3%, in line with the remaining spider genomes (lowest GC content *Latrodectushesperus* with 28.59% and highest content is *Stegodyphusmimosarum* with a GC content of 33.62; supplementary table 2, Supplementary Material online). The assembly has no obvious contaminants or associated symbionts, as determined by Blobtools (supplementary fig. 1, Supplementary Material online). The majority of scaffolds have a similar GC composition, despite variations in coverage. From all 3,925 scaffolds, 2,774 were labeled as no-hits (comprising only a total of ∼32.46 Mb of the assembly), and 889 labeled as Arthropods (∼886 Mb).

Annotation of the *T.kauaiensis* genome yielded 38,907 genes, comprising 213,695 exons and 171,423 introns (supplementary table 3, Supplementary Material online). Together, all genes cover 290,369,064 bp (290 Mb) representing 26.7% of the genome with 41,209,078 bp (41 Mb, 3.8% of the genome) being coding sequences (cds). The mean gene length is 7,463 bp (supplementary table 3, Supplementary Material online), the longest gene is 208,580 bp long (208 kb), and 89.7% of BUSCOs are retrieved as complete.

### Genome Characterization and Evolution

The analyzed assemblies vary widely in size. *Araneusventricosus* has the largest assembly with 3.6 Gb (supplementary table 2, Supplementary Material online), whereas *T. kauaiensis* has the smallest assembly with 1,085,571,486 bp (1.1 Gb). In between these extremes, we find the genomes of *S. mimosarum* (2.7 Gb), *Trichonephilaclavipes* (2.4 Gb), *Argiopebruennichi* (1.7 Gb), *Dysderasilvatica* (1.4 Gb), *Parasteatodatepidariorum* (1.5 Gb) and *L. hesperus* (1.1 Gb).

Considering the 3-fold variation in genome size and the evidence for ancient whole genome duplications in Chelicerata (Shingate et al. 2020) and Arachnida (Schwager et al. 2017; Harper et al. 2021), and the suggestion that there has been a large-scale (whole genome or chromosomal) duplication event within spiders (Clarke et al. 2015), we explored the possibility of whole genome duplication private to spider genomes by interrogating the number of homologs in the *Hox* genes clusters. Using *Hox* genes 1–5, and based on a threshold of 95% identity, we find no evidence for an additional ancestral whole genome duplication in the studied spider genomes. We found zero, one, or two homologs for *Hox* 1 (supplementary table 4, Supplementary Material online). For *Hox* 2, we found two homologs in all genomes, with the exception of *A*. *ventricosus*, where we only find a single homolog (supplementary table 4, Supplementary Material online). For *Hox* 3, there was only one homolog in all genomes, with the exception of *P. tepidariorum* (two candidates) and *T*. *clavipes* (no candidate). For *Hox* 4, we found two homologous genes in *T. kauaiensis*, *P. tepidariorum*, *L. hesperus*, and *S. mimosarum*, one in *T*. *clavipes* and another in *D. silvatica*. *A*. *ventricosus*, however, had four homologs for the Hox4 gene. Finally, for *Hox* 5, we identified one homolog in all genomes, with the exception of *A*. *ventricosus* and *P. tepidariorum* where we found two homologous genes. This suggests that, with the exception of the outlier with four copies (*Araneus* Hox4), *Hox* genes are present in one or two copies.

### Transposable Element Variation

**Fig. 1.**
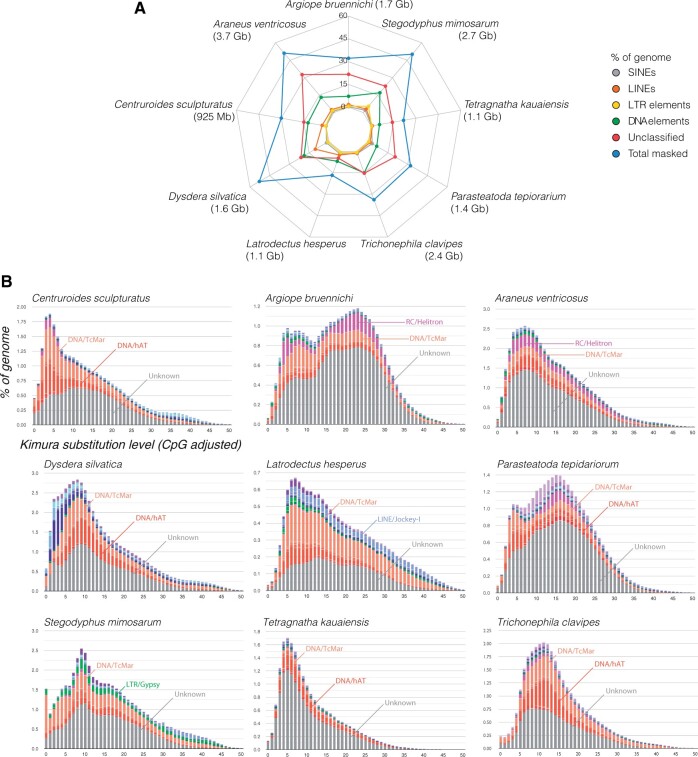
Transposable element and repeat characterization (*A*) Web diagram showing the representation of TE and repeats in the assemblies. Assemblies and correspondent assembly sizes are represented on the edges of the web diagram. Different transposable element families or repeats are presented in different colors on the plot, and the total content masked by RepeatMasker is shown in blue. The numbers for each web-line indicate the percent of the genome occupied by each transposable element, or the percent masked. (*B*) Repeat/transposable element landscape plots for the various assemblies. The three most represented transposable element categories are present for every genome (e.g. DNA/TcMar, DNA/hAT, and unknown for *T. kauaiensis*). Each plot shows the Kimura substitution level (*x* axis) and percent of genome covered by repeats (*y* axis).

We find variation in repeat content and tempo of repeat accumulation across the spider assemblies (fig. 1; supplementary table 5, Supplementary Material online). For example, 10.3% of the *D. silvatica* genome is composed of Long Interspersed Nuclear Elements (LINEs), whereas all other studied spiders had at most 3% LINEs (fig. 1*A*). *Stegodyphusmimosarum* had 5.40% of its genome covered by long terminal repeat (LTR) elements, whereas *A*. *ventricosus*, which is the second LTR-element-most rich genome, had only 1.60% (fig. 1). Interspersed repeats varied between 52.84% in *D. silvatica* and 16.53% in *L. hesperus* (supplementary table 5, Supplementary Material online). Unclassified repeats ranged between 32.64% (*A*. *ventricosus*), and 4.71% (*L. hesperus*) (supplementary table 5, Supplementary Material online). Overall, Repeatmasker identified between 16.71% and 52.84% of total repeat content (fig. 1*A*; supplementary table 5, Supplementary Material online). The correlation coefficient (*R*) between genome size and the percent of masked genome is *R* = 0.65, and the correlation coefficient (*R*) between total length of the masked genome and genome size is *R* = 0.962. Finally, we find variability in the accumulation of transposable elements through time, as represented by the shape of the transposable element/repeat landscape plot curves (fig. 1*B*). For instance, the *A. bruennichi* and *P. tepidariorum* assemblies show two peaks in transposable element accumulation, whereas all the others display a single peak. *Stegodyphusmimosarum*, however, has a recent burst in Tc1/mariner (DNA/TcMar) transposable elements (fig. 1*B*). Despite the differences in the accumulation of transposable element/repeats through time, we note that the Tc1/mariner group (DNA/TcMar) is present as one of the top three most represented transposable elements in all the assemblies, and hAT transposons (DNA/hAT) are also among the three-dominant categories in six assemblies. There is, however, variation across assemblies, as shown by the high numbers of Helitrons (RC/Helitron) in two of the Araneidae assemblies (*A. bruennichi* and *A*. *ventricosus*), Gypsy (LTRGypsy) in *S. mimosarum*, and Jockey (LINE/Jockey-l) in *L. hesperus*.

The analysis of genome completeness, as assessed by BUSCO scores, suggests that spider assemblies are considerably fragmented and missing substantial parts of the genome (supplementary table 6, Supplementary Material online). For instance, the *D. silvatica*, *L. hesperus* and *T*. *clavipes* genomes have only, respectively, 66%, 38.6%, and 52% complete BUSCOs (Arachnid odb10). Completeness in the remaining genomes ranged between 80% and 99%. Duplicated BUSCOs ranged between 30.5% (*P. tepidariorum*) and 3.2% (*S. mimosarum*). Notably, the two biggest genomes, *A*. *ventricosus* (3.6 Gb) and *S. mimosarum* (2.7 Gb) have 18.4% and 3.2% duplicated BUSCOs (supplementary table 6, Supplementary Material online, Arachnid data set odb10). The percentage of complete single-copy, duplicated, fragmented, and missing BUSCOs is concordant between the Arthropod and Arachnid sets (supplementary table 6, Supplementary Material online).

### Gene-Family Evolution

**Fig. 2.**
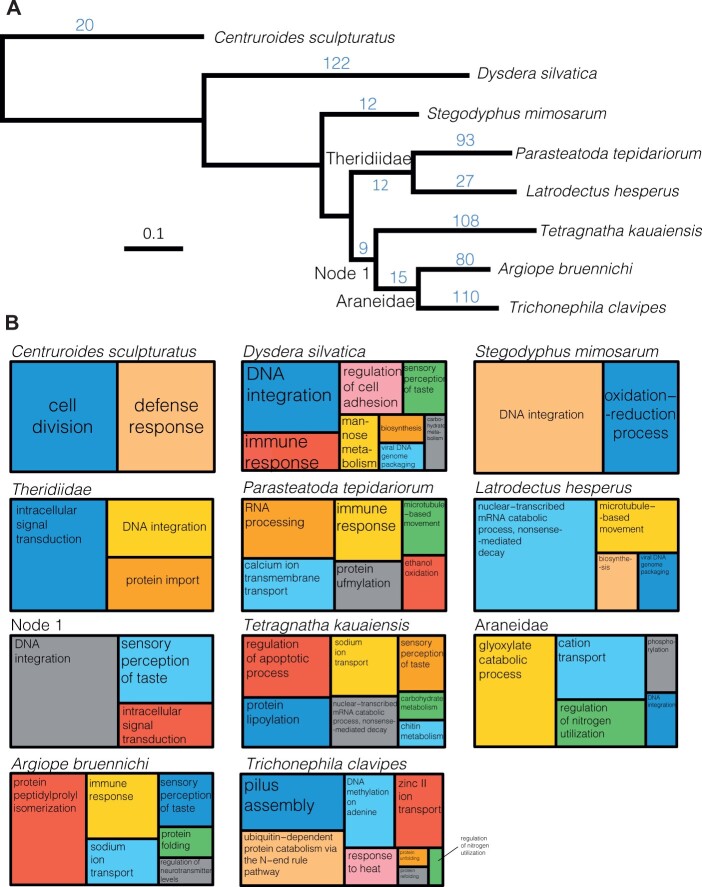
Gene family expansion (*A*) Tree topology obtained for single-copy orthologs. Numbers in blue indicate significantly expanded gene families as determined by CAFE. (*B*) Treemap representation of Gene Ontology Biological Function Annotation of the significantly expanded gene families as retrieved by REVIGO. Branches/Nodes with significant expansions, including Araneidae, Theridiidae, and Node 1 are represented together with the different genomes.

Because studying gene family evolution requires a phylogenetic backbone, we used the tree obtained from OrthoFinder based on 286 single-copy orthologs (orthologs are genes in different species that evolved from a common ancestral gene; fig. 2*A*). The tree topology has *T. kauaiensis* (Tetragnathidae) as sister lineage to the clade comprising the two members of Araneidae (*A. bruennichi* and *T*. *clavipes*). The clade encompassing all the aforementioned is sister to the Theridiidae (*L. hesperus* and *P. tepidariorum*). In turn, *S. mimosarum* (Eresidae) is the sister to Araneoidea (represented here by Tetragnathidae, Araneidae, and Theridiidae). *Dysderasilvatica* (Dysderidae) is the sister to the clade comprising all the aforementioned spiders (fig. 2*A*). This topology is in agreement with recent and comprehensive phylogenomic analyses of spiders (Fernández et al. 2018).

From a total of 608 significant gene family expansions in all branches, 572 occurred in terminal branches (fig. 2*B*). There were 451 significant expansions, and 157 significant contractions, of which 124 occurred in terminal branches (supplementary figs. 1–4, Supplementary Material online).

GO annotations of the significantly expanded gene families which were characterized under “biological process” were organized by REVIGO and are represented in fig. 2*B*. Broadly, we find expansions associated with feeding metabolism and sensory perception, mannose metabolism in the genome of *D. silvatica* and chitin metabolism in *T. kauaiensis* (fig. 2*B*). Expansions in carbohydrate metabolism are found in *D. silvatica* and *T. kauaiensis*, whereas Araneidae has glyoxylate catabolic process expanded (fig. 2*B*). Expansions in sensory perception of taste are found in *D. silvatica*, *T. kauaiensis*, *A. bruennichi*, and in Node 1 (fig. 2*B*). Immune response is found in the genomes of *D. silvatica*, *P. tepidariorum*, and *A. bruennichi*, whereas sodium ion transport is found in *T. kauaiensis* and *A. bruennichi* (fig. 2*B*).

When considering significant expansions in all GO categories (i.e. biological process, molecular function, and cellular component), we find expansions associated with taste (including sensory perception of taste in Node 1, *A. bruennichi*, and *D. silvatica*; detection of chemical stimulus involved in sensory perception of taste in *A. bruennichi* and Node 1; molecular function taste receptor activity is found in *A. bruennichi* and *T. kauaiensis;*supplementary table 7, Supplementary Material online). We also find evidence for expansions related to various metabolic processes, including carbohydrate metabolic process, and mannose metabolic process in *D. silvatica*, whereas protein catabolic process, 3,4-dihydroxybenzoate catabolic process, fatty acid catabolic process, pyruvate metabolic process, glucose metabolic process, protein metabolic process, lipid catabolic process, lipid metabolic process, and fatty acid metabolic process are found in *T*. *clavipes*. The *P. tepidariorum* genome includes expansions in peptidoglycan catabolic process and lipid metabolic process, whereas that of *T. kauaiensis* includes expansions in chitin metabolic process and carbohydrate metabolic process. Theridiidae includes expansions in lipid metabolic process, whereas Araneidae includes changes in taurine catabolic process. Finally, catalytic activity is expanded in the genomes of *D. silvatica*, *L. hesperus*, *T*. *clavipes*, *T. kauaiensis*. Other notable expansions include the regulation of neurotransmitter levels, structural constituent of eye lens in *A. bruennichi*, defense response and toxin activity in *C. sculpturatus*, and response to heat in *T*. *clavipes*. The biological process for “sodium channel activity” is found expanded in *A. bruennichi*, *T*. *clavipes*, and *P. tepidariorum*, whereas the molecular function for “sodium channel activity” is found in *A. bruennichi* and *T. kauaiensis*. Proteolysis (i.e. breakdown of proteins), the breakdown of process is expanded in *A. bruennichi*, *C. sculpturatus*, *D. silvatica*, *L. hesperus*, *P. tepidariorum*, *S. mimosarum*, *T. kauaiensis*, and Theridiidae.

### Venom Gene-Family Variation

**Fig. 3.**
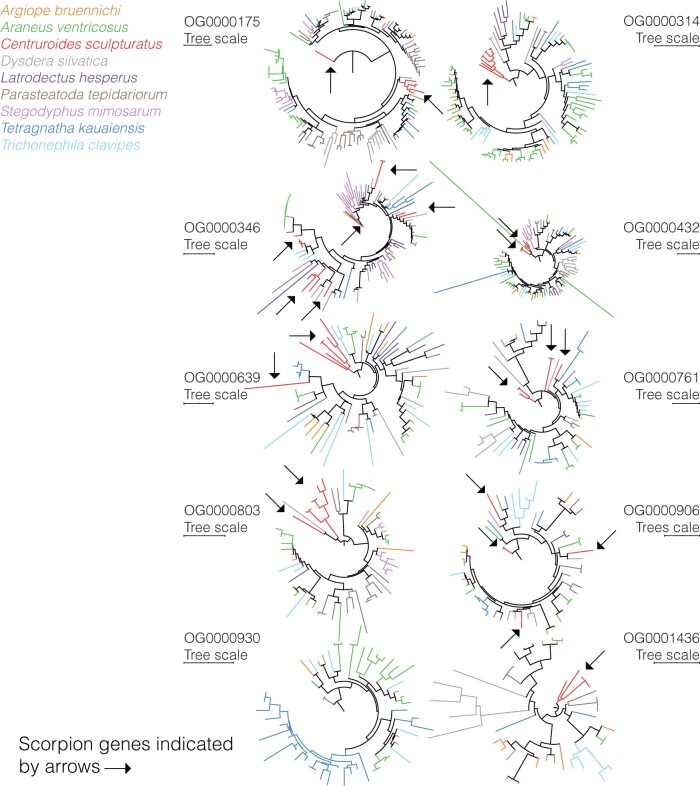
Venom gene phylogenies. Phylogenies for the ten largest orthogroups of identified venom genes. For each tree, we indicate the Orthogroup ID and tree scale. Different colors correspond to different species, as displayed in the legend. Arrows highlight scorpion toxin genes and show that most orthogroups were already present in before the split between scorpions and spiders.

The combination of BLAST and TOXIFY identified a total of 559 toxins in the studied genomes (supplementary table 8, Supplementary Material online), included as part of 189 orthogroups. The orthogroups with most genes are displayed in figure 3 and include OG0000175 (135 genes, Astacin-like metalloproteases as determined by NCBI-BLAST), OG0000314 (105 genes, Neprilysins or endothelin-converting proteins), OG0000346 (99 genes, uncharacterized proteins), OG0000432 (86 genes, Techylectin), OG0000639 (68 genes, various toxin-types), OG0000761 (61 genes, Zonadhesins, various toxin-types), OG0000803 (59 genes, Astacin-like metalloproteases), OG0000916 (54 genes, Papilins, Kunitz-type serine protease inhibitor) OG0000930 (54 genes, Astacin-like metalloproteases), OG0001436 (41 genes, uncharacterized proteins). The two most toxin-rich assemblies were the *A. bruennichi* and *P. tepidariorum* where 154 and 200 toxins were identified, respectively. The scorpion genome, *C. sculpturatus*, yielded 31 toxins, whereas *D. silvatica* and *L. hesperus* yielded 13 and 16 toxins, respectively (supplementary table 8, Supplementary Material online).

Phylogenetic analyses of the orthogroups show that most venom families were present before the split between scorpions and spiders (fig. 3). Different spider genomes include species-specific expansions (i.e. groups of five or more genes from a single genome that cluster as a monophyletic clade), and many of these have relatively large branch lengths. Specifically, we find evidence for various expansions in *P. tepidariorum* (4 expansions, one with 7 genes, another with 12, one with 7 and one with 9 genes), one expansion in *A*. *ventricosus* (one expansion with 11 closely related genes), one in *D. silvatica* (one expansion in 6 genes) and one in *C. sculpturatus* (5 genes expanded) in OG0000175 (fig. 3). In OG0000314, we found an expansion private to the three Araneidae genomes, including *A. bruennichi*, *A*. *ventricosus*, and *T*. *clavipes*), various expansions exclusive to the *A*. *ventricosus* genome, and one expansion specific to the scorpion genome (nine genes). In OG000346, we found various expansions on the *S. mimosarum* (nine genes), *P. tepidariorum* (five genes), *A*. *ventricosus* (eight genes) genomes. In OG000432 we found genome-specific expansions in *D. silvatica* (eight genes; fig. 3). In OG0000639, we found an expansion in *C. sculpturatus* (five genes), and in OG0000803 there are two five-gene expansions, one in *C. sculpturatus*, another in *A*. *ventricosus*. OG0000930 is only present in *T. kauaiensis* (1 expansion with 20 genes), *A*. *ventricosus*, *A. bruennichi*, *T*. *clavipes*, and *P. tepidariorum*. OG0001436 is expanded in *C. sculpturatus* (five genes).

### Chemosensory Gene-Family Variation

**Fig. 4.**
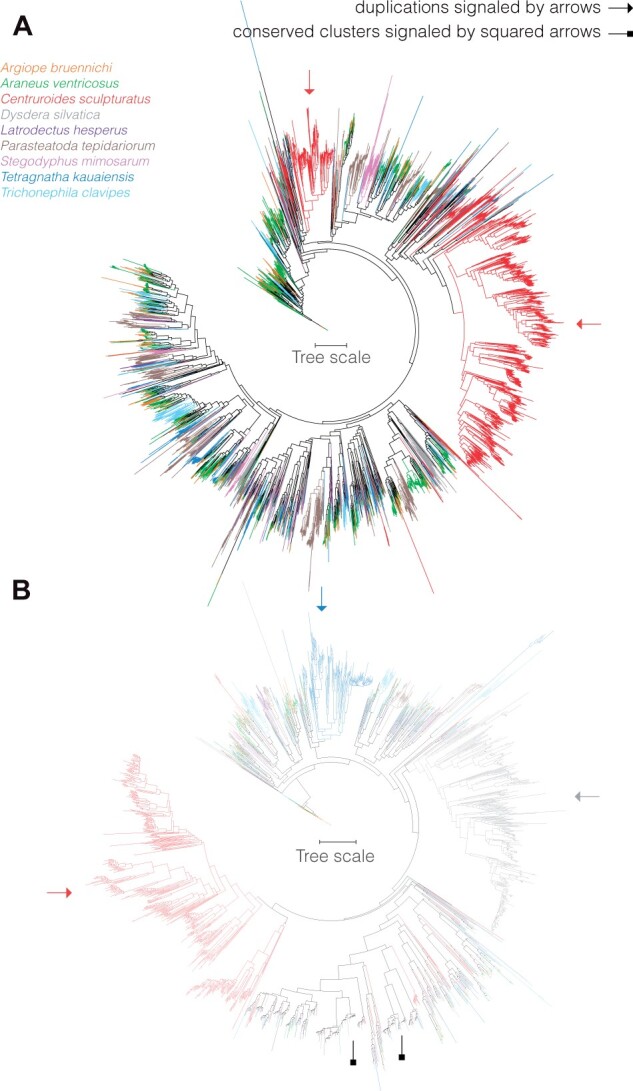
Gustatory and ionotropic reception phylogenies (*A*) Gustatory receptor phylogeny. The phylogeny has 5,595 genes and includes every GR identified in the assemblies herein studied. (*B*) Ionotropic receptor phylogeny. The phylogeny has 1,932 genes and includes every IR identified in the assemblies herein studied. Arrows indicate major duplications private to specific genomes, whereas squared arrows highlight potentially conserved IR genes (small branch length and small duplicates).

**Fig. 5.**
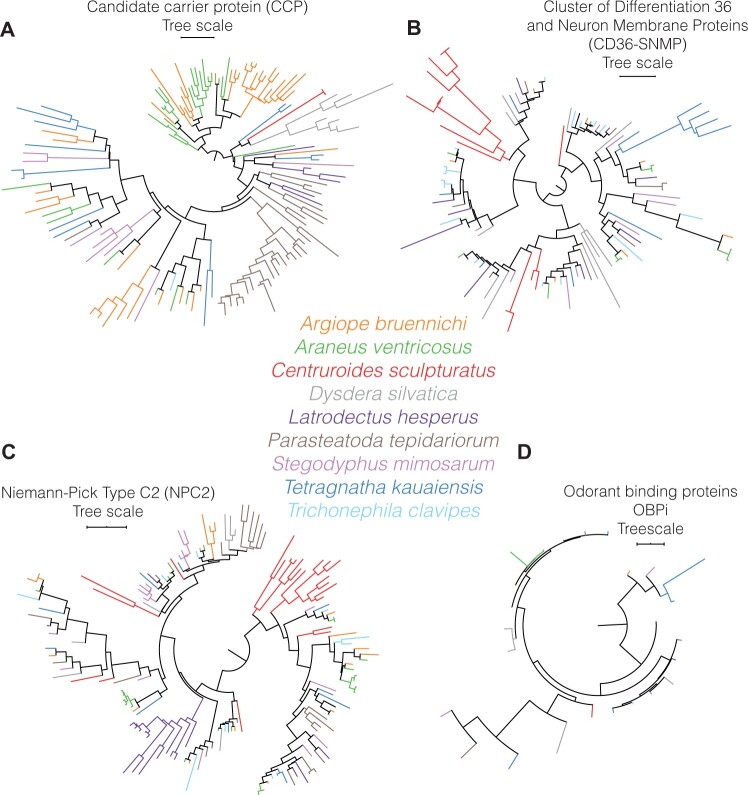
Phylogeny of other chemosensory genes. (*A*) CCP phylogeny; (*B*) CD36-SNMP phylogeny; (*C*) NPC2, phylogeny; (*D*) OBP-like phylogeny.

We identified a total of 5,595 candidate gustatory receptors (GRs), 1,934 candidate ionotropic receptors (IRs), 25 candidate Odorant binding proteins (OBP-like), 147 candidate Niemann-Pick type C2 (NPC2), 137 candidate carrier protein (CCP), and 998 candidate cluster of differentiation 36 and neuron membrane proteins (CD36-SNMP; supplementary table 9, Supplementary Material online; figs. 4 and 5). GRs exhibited a large interspecific variation (fig. 4), ranging between 1,436 GRs in *A*. *ventricosus* and 84 in *L. hesperus*. *Centruroidessculpturatus*, the outgroup, had 1,648 GRs (supplementary table 9, Supplementary Material online). The *D. silvatica* genome has the most IR/iGluR genes with 443 genes (supplementary table 9, Supplementary Material online; fig. 4). We detected a total of 25 OBP-like genes, with 5 being present in *T. kauaiensis*, 4 in *D. silvatica* and in *S. mimosarum*, 3 in *P. tepidariorum* and all remaining genomes having only 1 or 2 OBP-like genes (supplementary table 9, Supplementary Material online; fig. 5). From the 147 identified NPC2, *D. silvatica* had the least NPC2-genes (7 genes) and *A*. *ventricosus* the most (23). *Argiopebruennichi* had the most CCP, with 41 genes, whereas *C. sculpturatus* and *T*. *clavipes* had only 1 CCP (supplementary table 9, Supplementary Material online; fig. 5). Finally, we identified at least 8 and at most 16 CD36-SNMP genes. *T*. *clavipes* and *C. sculpturatus* had the most CD36-SNMP genes with 16 and 14, respectively, whereas *P. tepidariorum* and *A. bruennichi* had the least with 8 (supplementary table 9, Supplementary Material online).

An analysis of phylogenetic patterns suggests that the chemosensory portfolio is driven by a highly dynamic diversification process. For instance, within GRs there are two genome-specific expansions of genes in the scorpion, one including 1,237 genes and another 235 genes (fig. 4). A similar pattern is observed in the IRs where we find two genome-specific expansions private to the scorpion genome (88 genes, and 382 genes; fig. 4), a large genome-specific gene group with 392 genes in *D. silvatica*, and another in the *Tetragnatha* genome including 139 genes. In CCPs, we found expansions in *A. bruennichi* (5 genes and 13 genes), *P. tepidariorum* (21 genes), *A*. *ventricosus* (8 genes), and *D. silvatica* (6 genes; fig. 5*A*). In CD36-SNMP we found expansions in the scorpion (9 genes) and in *T. kauaiensis* (5 genes; fig. 5*B*). In NPC2, we found expansions in *L. hesperus* (14 genes), *P. tepidariorum* (6 genes), and *C. sculpturatus* (14 genes; fig. 5*C*), whereas in CD36-SNMP (fig. 5*D*) we found expansions in the *T. kauaiensis* (5 genes) and *C. sculpturatus* (9 genes) genomes.

### Silk Gene-Family

We identified a total of 24 putative spidroins in the genome of *T. kauaiensis* (supplementary table 9, Supplementary Material online). After querying these to the NCBI protein database, we identified one Flagelliform spidroin (Flag), four Aggregate spidroins (AgSp), eight Major Ampullate spidroins (MaSp), three Minor Ampullate spidroins (MiSp), one Tubuliform spidroins (TuSp), one Pyriform spidroin (PySp), and one Aciniform spidroin (AcSp). There was one spidroin for which NCBI did not yield any results, and four where the database retrieved more than a single gland as a top-hit (supplementary table 9, Supplementary Material online). Alignments are provided in the Supplementary Material online.

**Fig. 6.**
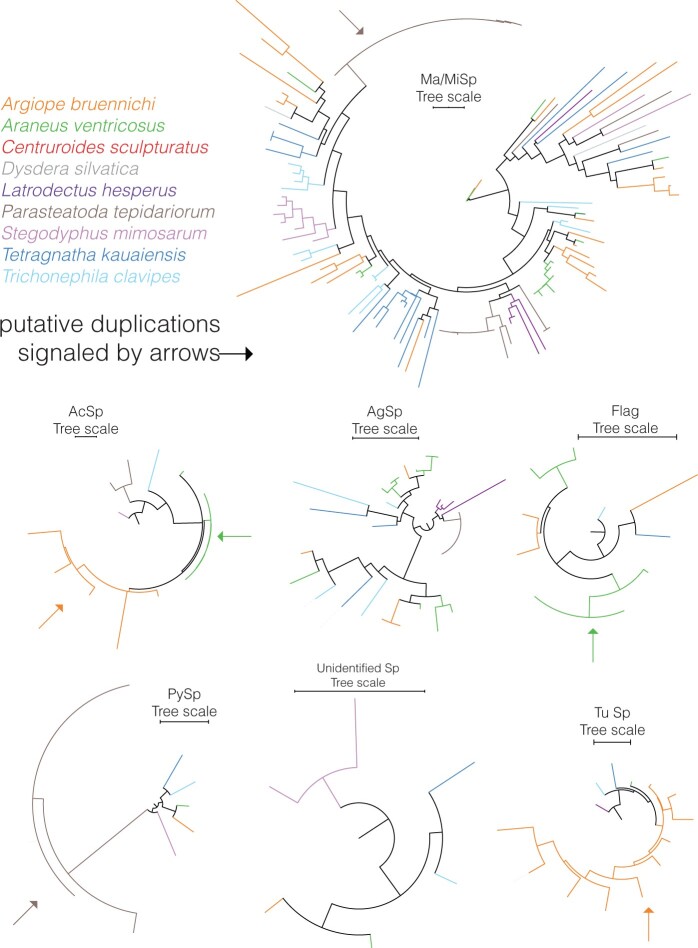
Silk genes (spidroins) phylogeny. These include Major and Minor Ampullate spidroins (Ma/MiSp), Aciniform spidroins (AcSp), Aggregate spidroins (AgSp), Flagelliform spridroins (Flag), Pyriform spidroins (PySp), an unidentified spidroins group present in the *Trichonephila clavipes* genome and the Tubuliform spidroins (TuSp).

Phylogenetic patterns of spidroin shows several genome-specific expansions of the Ma/Mi spidroins, including two separate expansions in the *P. tepidariorum* genome (25 genes and 10 genes; supplementary table 10, Supplementary Material online; fig. 6), a single expansion in *S. mimosarum* including 7 genes, another in *A*. *ventricosus* including 8 genes, and another in *T. kauaiensis* including 7 genes. In the remaining spidroins, we find genome-specific expansions in AgSp and PySp in *P. tepidariorum*, with nine and six genes, respectively. In AcSp there are two smaller lineage-specific clades in *A. bruennichi* and *A*. *ventricosus*. There is a genome-specific expansion in *A. bruennichi* for the TuSp gland, with seven genes (supplementary table 10, Supplementary Material online; fig. 6).

## Discussion

In this study, we report the sequence assembly of the *T.kauaiensis* genome, and explore genome evolution across the available spider assemblies. To do so, we controlled for the quality of the assemblies, by focusing on contiguity and completeness (i.e. how complete a genome is from a gene content perspective based on the presence of universal single copy genes), finding that many of these assemblies are highly fragmented and incomplete. We find a wide variation in gene content, repeat content, and genome size in the surveyed spider genomes, which indicates a highly dynamic pattern of genome evolution. Although the low quality of some assemblies did not hamper comparative analyses of the surveyed spider genomes, results should be interpreted with caution. By surveying all repeats and transposable elements (hereafter “the repeatome”) and studying *Hox* gene duplications, we find that the observed genome size differences are likely driven by the expansion of the repeatome. We also find significant gene-family expansions associated with sensory perception of taste, immunity, and metabolism, which may underlie the diverse biology of spiders. We confirm previous work showing that venoms and chemosensory genes are present in high numbers across the assemblies, and discuss the role of putative ancient whole genome duplication in generating the diversity we observe in spiders.

### Repeat Content Underlie Genome Size Variation in Spiders

Previous evidence from flow cytometry, Feulgen image analysis densitometry, and genome assembly sizes have found wide variation in genome size in spiders (Gregory and Shorthouse 2003; Sanggaard et al. 2014; Král et al. 2019). For instance, Gregory and Shorthouse (2003) assembled a large data set comprising 115 species from 19 different families of spiders, finding that spider genomes vary between 5.73 and 0.79 C (∼7 Gb for the jumping spider *H.borealis*—∼724 Mb for the long-jawed orbweaver *T.elongata*). They also reported a wide variation within relatively closely related species. For instance, genome size in the Salticidae family ranged between 1.73 and 5.73 C (between *H.borealis* and the peppered jumping spider *Pelegrina galathea*). Our results are in line with this evidence, because we found variation in genome size among spider assemblies (in our data set the largest genome was *A*. *ventricosus* with 3.6 Gb, and the smallest was *T. kauaiensis* with 1.08 Gb). We also report variation between relatively closely related species (i.e. within the Araneidae family, where we included three assemblies, genome sizes ranged between 3.6 and 1.7 Gb). Similar to previous reports, we do not find a clear phylogenetic pattern of genome size variation across the spider tree of life (Gregory and Shorthouse 2003).

Genome size may increase through whole genome duplication, where the whole genome doubles itself, or through small-scale duplication of genetic elements which may include duplication of genes or transposable elements. Recent evidence, using flow cytometry, has revealed a whole genome duplication in caponiid spiders (Král et al. 2019), which indicates the potential of further whole genome duplications in spiders, other than the duplication ∼450 Ma (Schwager et al. 2007, 2017). Although we have no caponiids in our data set, we found no evidence of recent whole genome duplication specific to spiders on the analyzed assemblies. This evidence comes from several sources. First, there is a low percent of double copy BUSCO genes—a set of highly curated genes, single copy genes. The scorpion assembly has a duplicate BUSCO score of 26%, whereas spider genomes range between 26% and 0.8%, in *P. tepidariorum* and *L. hesperus, respectively* (note that *L. hesperus* assembly has many missing BUSCOs, which is indicative of a poor assembly quality). Second, analysis of *Hox* genes shows that these genes are mostly present in two copies, with a single exception of four *Hox4* in *A*. *ventricosus*. The four copies of *Hox4* in *A*. *ventricosus* could be an artifact due to the similarity between Hox genes, and we were not able to obtain candidates for *Hox1* using the 95% cut-off threshold. The BUSCO-pattern together with that from the *Hox* genes is in line with the evidence for ancestral whole genome duplication in Arachnopulmonata. Third, an important finding of our work is that variation in genome size of spiders is largely driven by the duplication of genetic elements, and specifically, the repeatome (transposable elements and repeats). Indeed, we find an *R* = 0.95 correlation between the “length of the masked repeats” and the “genome size”—a strong indication of the role of the repeatome in underlying genome size changes (fig. 1). Expansions of the repeatome are generally constrained in animal lineages because bigger genomes translate to higher cell-economy costs through the increase of cell size. In addition to this, proliferation of transposable elements may interfere with gene expression when these selfish elements jump in front of a gene promoter (Choi and Lee 2020). Considering the strikingly different representation of the repeatome that we find here, including the variation in transposable element accumulation through time, we speculate that transposable elements may have had a role in the regulation and variation of gene expression across spiders, likely underlying some of the observed morphological and physiological diversity.

By conducting a de novo annotation of repeats and using the same version and library of repeats for every genome, we guaranteed a standardization of the repeat identification, thereby removing potential biases due to the use of different databases and pipelines. Variation in some elements, both in terms of classes and extent along the genome, was substantial. For instance, LINESs represent less than 2% in every assembly, but represent 10.3% of the *D. silvatica* assembly. This may suggest mechanisms to purge LINEs from some clades, or an expansion specific to *D. silvatica* (and possibly closely related species). Furthermore, DNA elements had a 3-fold variation, ranging between 5.59% (*T. kauaiensis*) and 18.82% (*D. silvatica*). Despite the overall variation in numbers and accumulation of the repeatome through time, there was a clear dominance of DNA/TcMar and DNA/hAT elements (both DNA elements) across the assembly when considering the top three most represented categories (fig. 1*B*), suggesting these elements are the most prolific and present across spiders, and potentially scorpions (keep in mind we have single scorpion genome in our analyze using the same version and library of repeats for every genomes). Future studies on spider genome assemblies should put transposable element variation in the context of the spider phylogeny, and should benefit from an increased sampling of spider genomes. The differential presence of repeats and transposable elements may indicate that mechanisms to eliminate these elements such as nonhomologous end joining or illegitimate recombination may be active in these genomes (Choi et al. 2020). A phylogenetic framework together with ancestral character reconstructions, focusing on transposable element data, will certainly elucidate the patterns of activation and deactivation of certain transposable element classes, and how changes in transposable element proliferation may be linked to particular events in the evolution of spiders. For instance, a caponiid genome, where a more recent genome duplication was detected (Král et al. 2019), may help understand the impacts of whole genome duplication and transposable element proliferation in spiders. This would allow testing the “genomic shock” hypothesis after genome duplication in spiders. Finally, the variation in the repeatome is in line with those of the remaining arthropods, where variation in transposable elements load was deemed as an important predictor for genome size (Wu and Lu 2019; Gilbert et al. 2021).

### Gene Duplicates

Observed patterns in the explored gene families, namely venoms and chemosensory, suggest a central role in the evolution of spiders (figs. 3–5). The presence of most gene families in the scorpion genome and in spider genomes suggests an ancestral status (Vizueta, Escuer, et al. 2020), whereas variation in gene numbers and their branch lengths along the phylogeny is an indication of divergence, and thereby indirect evidence of the acquisition of novel gene functions (i.e. neofunctionalization). Gene duplicates generally experience relaxation of purifying selection or gene dose compensation and, if one of the copies does not get sub- or neofunctionalized through time, it will be lost. Indeed, we manually curated chemosensory genes, finding a low ratio of pseudogenes (supplementary table 9, Supplementary Material online). There are large genome-specific duplications detected in *C. sculpturatus*, *T. kauaiensis*, and *D. silvatica* in the two largest chemosensory families (fig. 4*A* and *B*). This is an indicator of the importance of GRs and IRs in *T. kauaiensis* and *D. silvatica*, and we speculate it may be associated with the colonization of islands (*T. kauaiensis* is part of a Hawaiian radiation of spiders, and *D. silvatica* is part of a Macaronesian radiation) where environmental conditions can be very different (disharmonic biotas, open ecological niches) (Vizueta et al. 2019). We note that, unfortunately, the taxonomic range (i.e. one single genome for Tetragnathidae and one single for Dysderidae) does not allow dissecting whether these changes are shared by other members of the families, whether they are private to the species in question (*D. silvatica*, *T. kauaiensis*) or even to the adaptive radiations (occuring in Hawai’i and Macaronesia). Similarly, because we only included a single scorpion assembly, we cannot comment on whether the expansions observed in *C. sculpturatus* are specific to all scorpions, or just the *C. sculpturatus* genome.

Despite the aforementioned evidence, not every gene family is present in very high numbers. For example, we detected only 25 OBP-like genes in all genomes, and the small number of genes together with the short branch lengths confirms that the OBP-like are a relatively conserved family of genes in arachnids (Vizueta et al. 2017). In addition to the OBP-like, we also find few silk genes, with very short branch lengths (notice *P. tepidariorum* in PySp and Ma/MiSp, *A*. *ventricosus* in Flag and AcSp), which may be indicative of very recent duplications in silk genes (Garb et al. 2007; Clarke et al. 2014, 2015). These results are in line with those of Clarke et al. (2015) who used transcriptomics to suggest that a large-scale duplication occurred early in the divergence of spiders, and that multiple independent duplication events in silk genes have likely taken place afterwards. Our results, however, have to be interpreted with caution because silk genes are composed of sequences (of often hundreds) of repeated aminoacids (Clarke et al. 2015), being therefore hard to reconstruct in entirely in the gene annotation process, and being typically fragmented onto separate fragments. Considering the fragmentation of most assemblies, it is possible that some duplicates consist of gene fragments.

### Significant Expansion of Metabolism, Immunity, and Sensory Perception Gene Families

Using a statistical approach to detect expansion of gene families, we find that most expansions are in terminal branches. As a direct comparison, recent analyses on 76 insect assemblies were able to identify 147 expanded gene families, comprising 9,601 genes, in the branch corresponding to insects (“the Last-Insect-Common-Ancestor”; Thomas et al. 2020), thereby providing evidence for “ancient expansions” particular to insects. Thomas et al. (2020), however, included ten times more genomes than we did, and some of the spider genomes in our data set lack substantial data, as indicated by the BUSCO scores (supplementary table 6, Supplementary Material online). Thus, it is possible that spiders have their own set of “ancient expansions,” which we were not able to detect due to the limitations of our data set. It is also possible that the inclusion of fragmented assemblies (*D. silvatica* and *L. hesperus*) leads to an inflation of expanded gene families on closely related assemblies (e.g. *Parasteatodatepidariorum*). We expect that the addition of more highly completed spider genomes will help to further our understanding of the evolutionary history of gene families in spiders.

Despite the challenges in the data set, we find notable evidence for various gene families expansions in spiders. Specifically, using GO annotations we find that gene families associated with various metabolic functions, sensory perception of taste, and immune functions are expanded. This pattern is similar to the pattern found in arthropods which includes expansions of metabolic genes (Thomas et al. 2020). These independent pieces of evidence suggest that gene duplications associated with metabolism, immunity, and sensory functions may have been instrumental to the evolution of arthropods in general, but also spiders specifically. We speculate that these expansions may contribute to the success, in terms of number of species and adaptation to different environments in spiders. As chromosome resolved assemblies become cheaper and technically less challenging, revising the role of gene expansions and gene contractions will certainly yield important insights toward the understanding of genome evolution of spiders.

## Conclusion

We have sequenced the *T.kauaiensis* genome, and explored patterns of genome evolution across various genome assemblies. Comparative genomics analyses including *T. kauaiensis*, one scorpion (outgroup), and seven additional spiders assemblies suggest that variation of transposable elements and repeat content are associated with the wide variation of spider genome sizes. We also found many duplications in chemosensory and venom genes, consistent with the evidence that the evolution of toxins and the ability to perceive the environment are ancestral attributes of spider evolution. Our results suggest that the evolutionary history of spiders is characterized by gene-family expansions associated with sensory perception of taste, metabolism, and immune responses, and by multiple gene duplication events. Although we uncovered interesting patterns of genome evolution, we acknowledge the limitations of this work due to the lack of high-quality genomes. We hope that, however, this work catalyzes enthusiasm in the spider research community to produce and analyze more high-quality genomes.

## Materials and Methods

### *Tetragnatha kauaiensis—*Genome Sequencing, Assembly, Annotation, and Quality Verification

We sequenced the genome of a single individual of *T. kauaiensis* using a paired-end and a non-size selected mate-pair library on a lane of Illumina HiSeq4000 (individual ID AJR402, collected May 31, 2013 by AJ Rominger in Kaua’i, at 22.1412, –159.6206). Using these libraries we built a base assembly using ALLPATHS-LG with default parameters in addition to “HALOIDIFY = True” (Gnerre et al. 2011). We then sequenced an additional individual using the Dovetail Chicago method (AJR443, collected June 3, 2013 by AJ Rominger in Kaua’i, at 22.1469, –159.6638), which was used to scaffold the initial assembly using the HiRise software (Koch 2016; Putnam et al. 2016).

The quality of the assembly was first assessed using BUSCO v3.0.2 arthropoda db v9 (Simão et al. 2015), which searches for highly conserved genes in the assembly. Then we used the Assemblathon 2 script (https://github.com/ucdavis-bioinformatics/assemblathon2-analysis) (Bradnam et al. 2013), which assesses scaffold and contig statistics, to evaluate the quality of the assembly. Annotation of repeats was carried out by identifying and building a database of repeats along the genome using RepeatModeler followed by masking them using RepeatMasker (Tarailo-Graovac and Chen 2009). We explored the draft assembly for contaminants, including gut-microbiota and wet-lab contaminants using Blobtools (Koutsovoulos et al. 2016; Laetsch and Blaxter, 2017) (supplementary fig. 1, Supplementary Material online).

To determine protein-coding genes and their locations along the genome, we used BRAKERv1 (Hoff et al. 2019). We used whole-body *T. kauaiensis* transcriptome reads previously generated by Yim et al. (2014) (SRR1313313, SRR1427109). Raw transcriptomic reads were cleaned using Trimmomatic (Bolger et al. 2014) and aligned to the generated genome using STAR (Dobin et al. 2013). The resulting binary alignment map file was provided to BRAKERv1 as RNA-based evidence. The final annotation was assessed by BUSCOv4.0.1 (Seppey et al. 2019), using the Arthropoda10 (1,013 genes) and Arachnida10 (2,943 genes) gene sets.

### Genomes Used for Comparative Genomics

We searched the I5K and NCBI databases and the literature for published and available spider genomes (data consulted on October 23, 2019). In total, we downloaded nine spider genomes (supplementary table 1, Supplementary Material online), their general feature format (gff3), and predicted protein files (faa; supplementary table 1, Supplementary Material online). From the available genomes, we selected those with a contig-N50 above 8,000 bp in order to avoid genomes that were highly fragmented. This included the genomes of *S.mimosarum* (Sanggaard et al. 2014), *L.hesperus* (BCM-HGSC website), *P.tepidariorum* (Gendreau et al. 2017), *T.clavipes* (Babb et al. 2017), *D.silvatica* (Sánchez-Herrero et al. 2019), *A.ventricosus* (Kono et al. 2019) and *A.bruennichi* (Sheffer et al. 2021). Additionally, we downloaded the genome of the bark scorpion *C.sculpturatus* (Schwager et al. 2017) as an outgroup.

### Characterization of Spider Genomes

We characterized spider genomes based on the 1) continuity and completeness of the assemblies, 2) assembly size, 3) repeat-content, and 4) broad genomic features. Specifically, 1) the continuity of each genome serves as a proxy of the overall quality of an assembly, and it affects the detection of genes, repeat sequences, and transposable elements (Peona et al. 2018). We characterized the contiguity of the assemblies using the Assemblathon 2 script, as described above for *T. kauaiensis*, retrieving contig-N50, scaffold-N50, total number of contigs, total number of scaffolds, maximum scaffold size, assembly size, and GC content. 2) The “completeness” of the assemblies is generally defined as an overview of the genes which may be missing, fragmented, duplicated, or present in a single copy in an assembly. To assess the completeness of the genomes, we used BUSCO v4.0.1 as outlined above for *T.kauaiensis* (the Arthropoda10 set including 1,013 genes; and the Arachnida10 set including 2,943 genes). 3) To assess repeat content, we used Repeat-Modeler v2.0.1 and Repeat-Masker-v4.1.0. Repeat content in the genome includes simple repeats (typically 1–5 base pairs, e.g. AAA, TTTTT), tandem repeats (100–200 base pairs), segmental duplications (10,000–300,000 base pairs), and interspersed repeats (SINES, which are nonfunctional copies of RNA genes that were reintegrated into the genome; DNA transposons; LINES, which are non-retrovirus retrotransposons). We ran RepeatModeler and RepeatMasker for each genome to screen and annotate DNA sequences de novo, thereby annotating and masking repeats. We retrieved repeat-statistics including percent of the genome covered by different repeats and transposable element landscape plots. Finally, 4) we assessed broad genomic features including, among others, the number of genes, coding sequences, introns, gene length using Another Gff Analysis Toolkit v0.4.0 (AGAT available at https://github.com/NBISweden/AGAT/; agat_sp_functional_statistics.pl, and agat_sp_statistics.pl). The association between total genome size, and percent of masked sequences and total length of masked genome was assessed with a correlation using the cor() function in R.

### Spider Genome Evolution

Previous work suggests that the whole genome duplication in the common ancestor of scorpions and spiders can be linked to the diversification of spiders (Schwager et al. 2007, 2017). To better understand the presence of whole genome duplication in the studied lineages, we used two complementary approaches. We first analyzed repeat content variation in the available spider genomes (as described above), because differences in repeat content may translate to differences in genome size. Second, we downloaded the *Hox* genes 1–5 from the *P. tepidariorum* genome, and searched for these in the remaining spider genomes using BLAST (Altschul et al. 1990). *Hox* gene-copies are prime candidates for detecting whole genome duplications because they are functionally constrained (Leite et al. 2018). For example, a 1:4 ortholog ratio is maintained between the *Drosophila melanogaster* genome and vertebrate genomes, indicating the two whole genome duplications that occurred in the lineage of modern vertebrates (Hakes et al. 2007; Schwager et al. 2017).

### Spider Gene-Family Evolution

Another component of genome evolution is gene-family expansion and reduction, or the gain and loss of gene-copies. Focusing on the predicted-proteins resulting from the annotations of the spider genomes, we first cleaned and filtered sequences using Kinfin’s filter_fastas_before_clustering.py (Laetsch and Blaxter 2017) removing sequences shorter than 30 amino acids. We then removed all isoforms, keeping only the longest isoform using in-house scripts. For this analysis, we removed the genome of *A*. *ventricosus* since it has twice the number of genes compared with the other spider genomes, and this biases the analysis. Cleaned and isoform-free prediction-proteins were then analyzed using Computational Analysis of Family Evolution (CAFE v 4.2.1) (De Bie et al. 2006). Briefly, we first determined gene-similarity (based on BLAST *e* values) in the data set using an all-by-all BLAST approach. We then applied a Markov Cluster algorithm (MCL; mcxload, mcl mcxdump) (Enright et al. 2002), and parsed the output using the mcl2rawcafe.py script. These clusters (gene-families) are then integrated in a phylogenetic-backbone, which was retrieved from OrthoFinder’s single-copy orthologs (Emms and Kelly 2015). This tree was then converted to an ultrametric format with r8s (Sanderson 2003), using the divergence time of 175 Myr between Tetragnathidae (*T. kauaiensis*) and Araneidae (*A. bruennichi*) as a calibration point (Fernández et al. 2018). We used Dendroscope’s Graphical User Interface to visualize trees and remove bootstrap support (Huson and Scornavacca 2012). Using the main pipeline of CAFE, we estimated the birth-death parameter lambda (λ = 0.0021) for the data set and obtained information on gene-family under significant evolution.

Genes belonging to gene-families that have undergone significant changes, that is, fast evolving families, were annotated using GO terms using the command-line version of Interproscan v5.34–73.0 (Ashburner et al. 2000). GO term annotations for genes belonging to expanded or reduced gene families were summarized and plotted as a treemap using R (R Core Team 2013) with REVIGO’s treemap script (Supek et al. 2011).

### Silk, Chemosensory, and Venom Gene Variation

To investigate venom gene evolution, we downloaded all toxin sequences available in the Arachnoserver v3.0 (Pineda et al. 2018), and used these as a database to query proteins from the spider and scorpion genomes with BLAST. Hits with *e* values below 1e–10 were considered as candidate venom-genes. However, because venom proteins are potentially highly divergent and typically short, BLAST searches may result in a high proportion of false positives. To address this issue, we ran TOXIFY on the candidates, a pipeline specifically designed to identify toxins using deep learning algorithms (Cole and Brewer 2019). TOXIFY generates a prediction score between 0 and 1 where the higher the score, the more likely a molecule is to be a venom, and we selected values above 0.75 as a criterion here. After TOXIFY, we kept a list of 589 putative venom genes across the assemblies. We then used OrthoFinder, obtaining an orthogroup-assignment for each of these 589 venom genes, finding that they group in 189 orthogroups. From these 189 groups, we selected the 10 biggest (in terms of gene number), identified the toxin-group using NCBI nr protein database, and aligned the genes within orthogroups using mafft v7.455 (Katoh and Standley 2013). These alignments were then used to obtain a maximum likelihood phylogenetic tree with bootstrap estimate (automatic determination of the substitution model) using IQ-Tree v1.6.12 (Nguyen et al. 2015; Chernomor et al. 2016; Kalyaanamoorthy et al. 2017; Hoang et al. 2018). The resulting phylogeny was plotted, formatted, colored, and labeled using the iTOL web server (Letunic and Bork 2019).

Considering the recent evidence on the wide variation in chemosensory gene-family size in Chelicerates (Vizueta et al. 2017, 2018), we searched the available genomes for GRs, IRs, NPC2, OBP-like, CCP, CD36-SNMP. To do so, we used BITACORA v1.2 (Vizueta, Escuer, et al. 2020; Vizueta, Sánchez-Gracia, et al. 2020), using its GeMoMa algorithm (Keilwagen et al. 2019), benefiting from a curated chemosensory database used in Vizueta et al (2018). To ensure the quality of the annotations, we performed a round of manual curation of the results, guaranteeing that 1) only a single isoform was selected and 2) that putative annotation artifacts including small fragments, chimeric annotations, or identical proteins by misassembly of duplicated contigs were removed. Finally, curated gene members were classified as pseudogenes (i.e. sequences with in-frame stop codons), partial or putatively complete functional proteins. The identified GRs, IRs, NPC2, OBP-like, CCP, and CD36-SNMP were aligned using mafft, and a tree was generated and plotted using IQ-Tree and iTOL as described above.

We next identified spidroins (silk genes). To do so, we used a combination of BLAST searches using N-domains published with the *T*. *clavipes* genome, and the NCBI accession numbers for N-terminals and C-terminals from Vienneau-Hathaway et al. (2017). We extracted hits with an *e* value below 1e–10 and candidate silk genes were then queried in NCBI nr database search (blastp) to classify the gland to which they belong based on NCBI’s top hit. After labeling the gland, we did an orthogroup assignment using OrthoFinder as described above, and built a phylogeny for the silks in each gland, using the same method as described above for venom genes.

## Supplementary Material

Supplementary data are available at *Genome Biology and Evolution* online.

## Supplementary Material

evab262_Supplementary_Data
