# Supplementary material for: The Tetragnatha kauaiensis Genome Sheds Light on the Origins of Genomic Novelty in Spiders
Source: Genome Biol Evol. 2021 Nov 26;13(12):evab262. doi: 10.1093/gbe/evab262 (PMC8693713; doi:10.1093/gbe/evab262)
Supplement: evab262_Supplementary_Data [file evab262_supplementary_data.zip › supplementary_Figures_Merged.pdf]

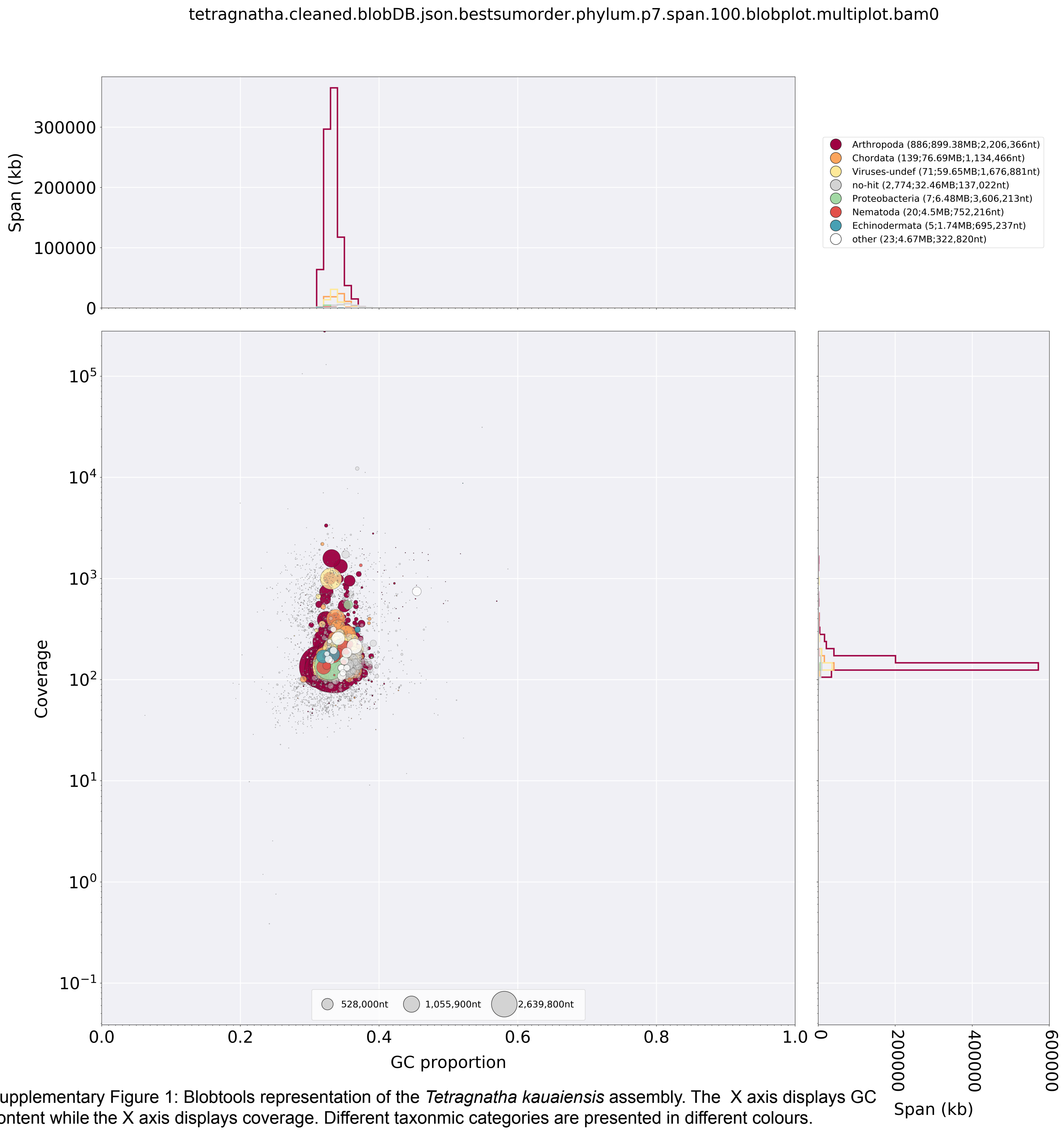

Supplementary Figure 1: Blobtools representation of the *Tetragnatha kauaiensis* assembly. The X axis displays GC content while the X axis displays coverage. Different taxonmic categories are presented in different colours.

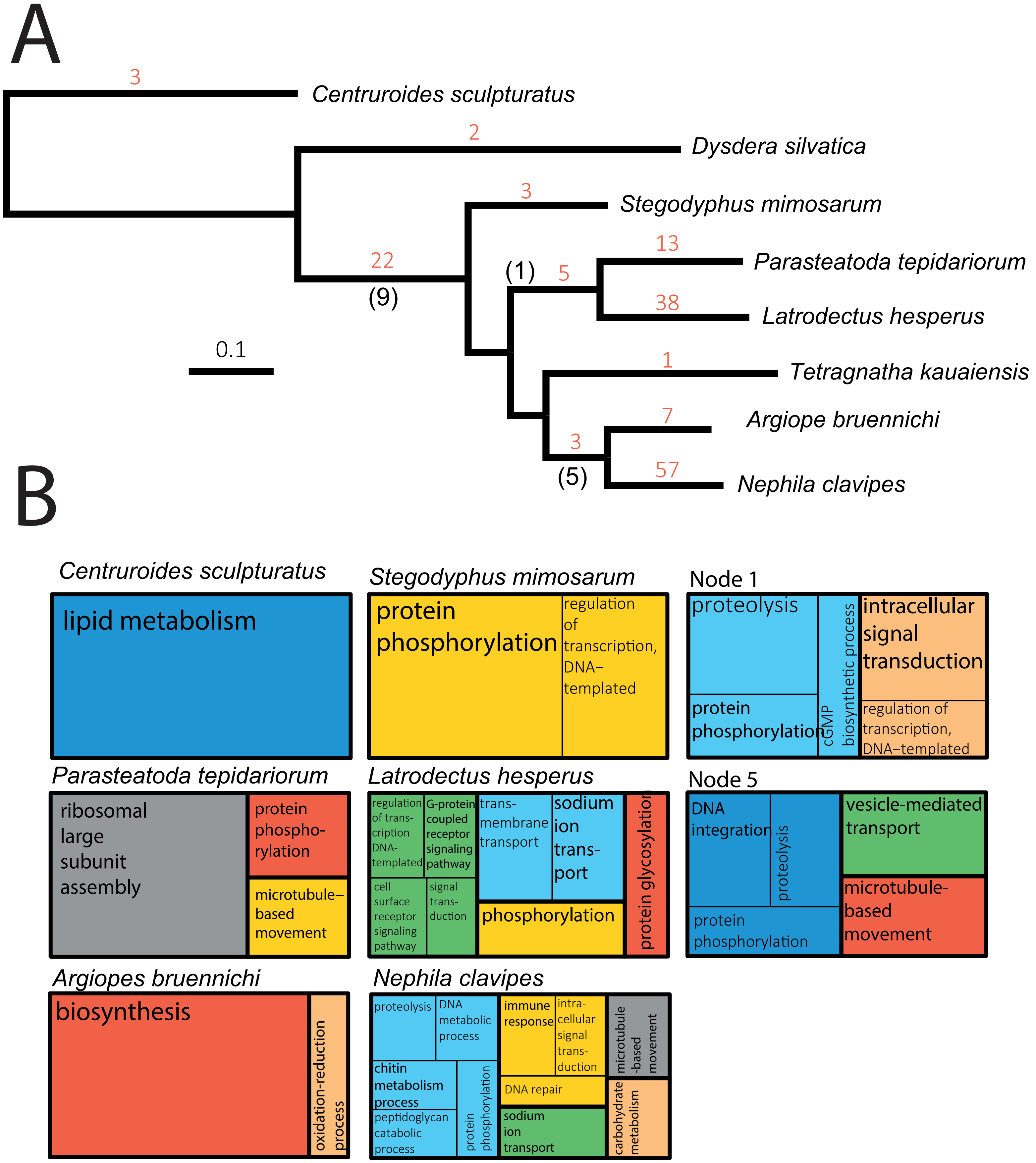

Supplementary Figure 2: Contracted gene families in spider genomes. A) Tree topology obtained from single-copy orthologs. Black numbers between (brackets) indicate branch/node ID, while numbers in red indicate significantly contracted gene families as determined by CAFE. B) Treemap representation of Gene Ontology **Biological Function Annotation of the significantly contracted gene families** as retrieved by REVIG Branches/Nodes with significant contractions are represented together with the different genomes.

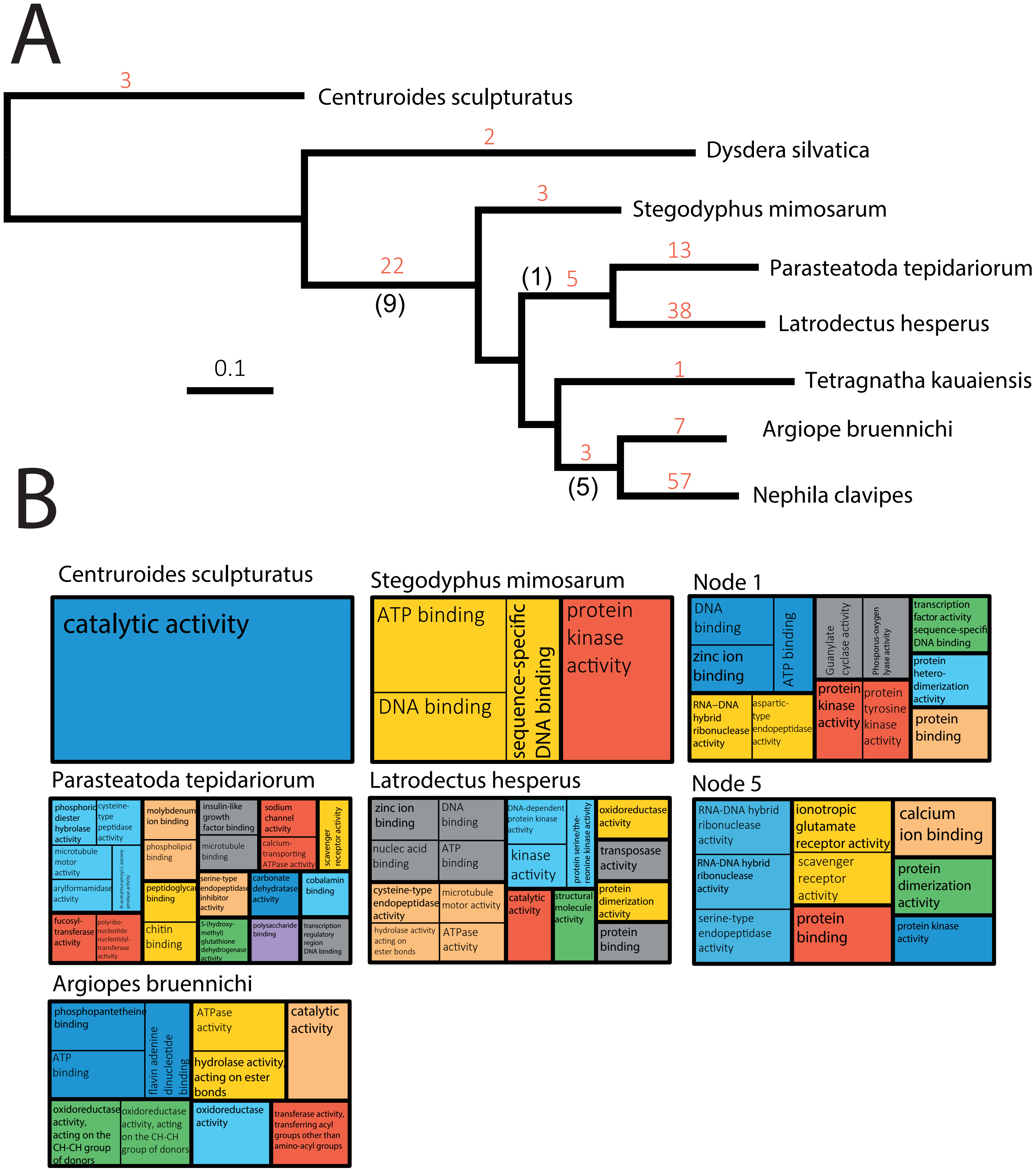

Supplementary Figure 3: Contracted gene families in spider genomes. A) Tree topology obtained from single-copy orthologs. Black numbers between (brackets) indicate branch/node ID, while numbers in red indicate significantly contracted gene families as determined by CAFE. B) Treemap representation of Gene Ontology **Molecular Function Annotation of the significantly contracted gene families** as retrieved by REVIGO. Branches/Nodes with significant contractions are represented together with the different genomes.

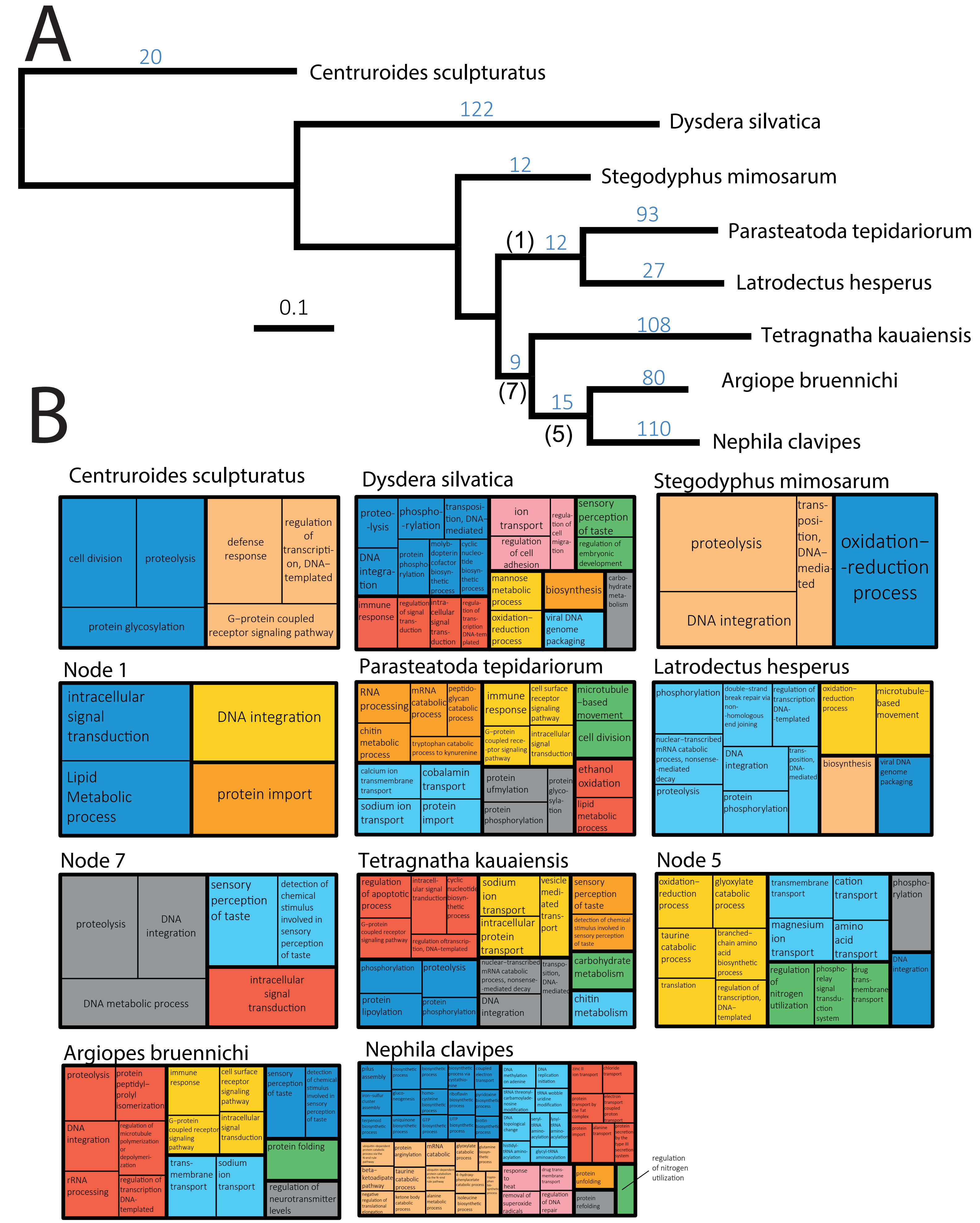

Supplementary Figure 4: Expanded gene families in spider genomes. A) Tree topology obtained from single-copy orthologs. Black numbers between (brackets) indicate branch/node ID, while numbers in blue indicate significantly expanded gene families as determined by CAFE.
B) Treemap representation of Gene Ontology **Biological Function Annotation of significantly expanded gene families** as retrieved by REVIGO. Branches/Nodes with significant expansions are represented together with the different genomes.

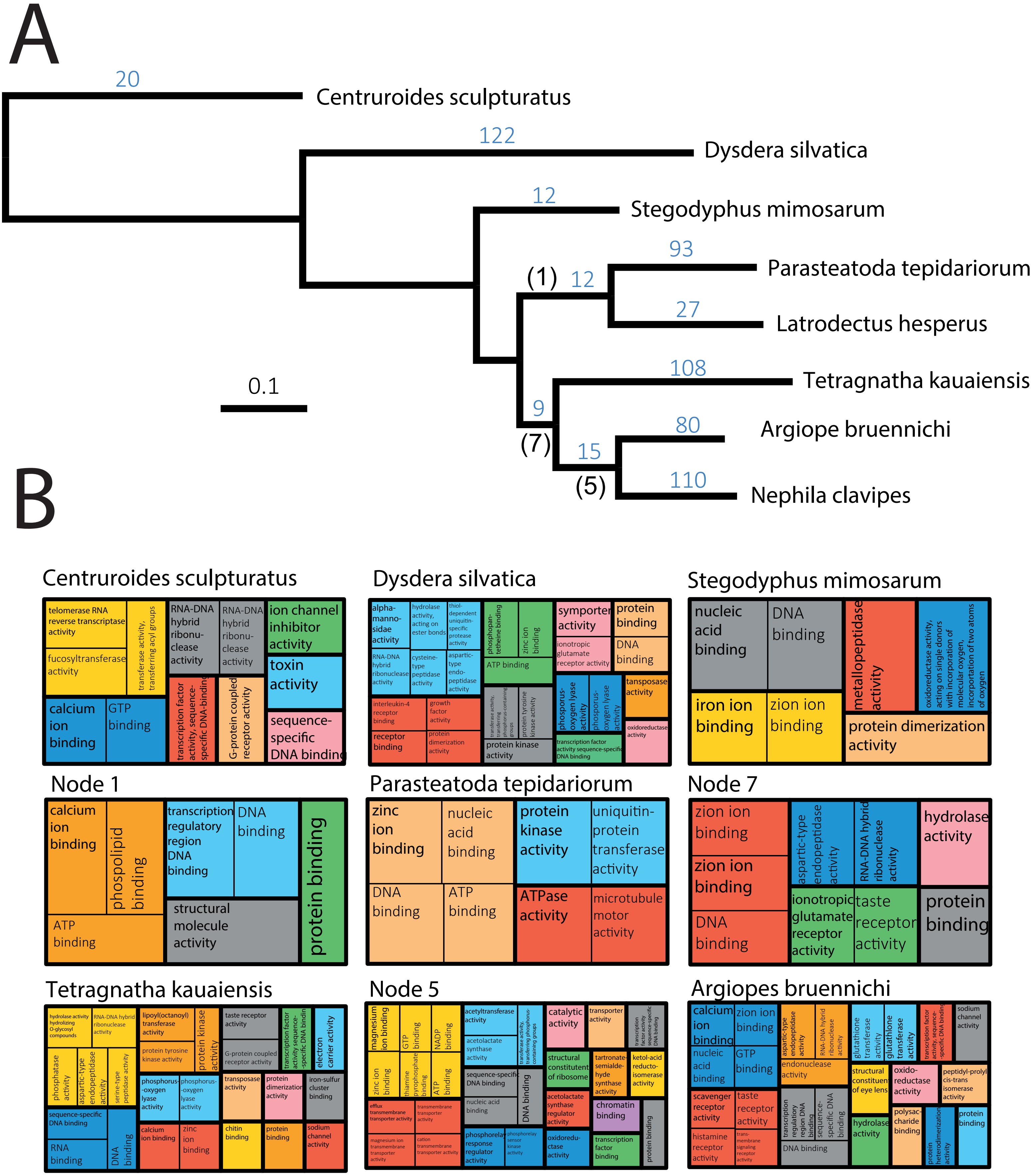

Nephila clavipes

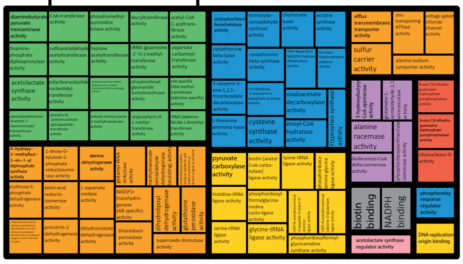

Supplementary Figure 5: Expanded gene families in spider genomes. A) Tree topology obtained from single-copy orthologs. Black numbers between (brackets) indicate branch/node ID, while numbers in blue indicate significantly expanded gene families as determined by CAFE.
B) Treemap representation of Gene Ontology **Molecular Function Annotation of the significantly expanded gene families** as retrieved by REVIGO. Branches/Nodes with significant expansions are represented together with the different genomes.
